# Antibiogram Profile and Detection of Resistance Genes in *Pseudomonas aeruginosa* Recovered from Hospital Wastewater Effluent

**DOI:** 10.3390/antibiotics12101517

**Published:** 2023-10-06

**Authors:** Joan U. Okafor, Uchechukwu U. Nwodo

**Affiliations:** Patho-Biocatalysis Group (PBG), Department of Biochemistry and Microbiology, University of Fort Hare, Private Bag X1314, Alice 5700, South Africa; okaforjoan14@gmail.com

**Keywords:** wastewater, antibiogram, *P. aeruginosa*, environment, antibiotics resistance

## Abstract

The nosocomial pathogen *Pseudomonas aeruginosa* (*P. aeruginosa*) is characterized by increased prevalence in hospital wastewater and is a public health concern. Untreated wastewater severely challenges human health when discharged into nearby aquatic ecosystems. The antibiogram profiles and resistance genes of *P. aeruginosa* were evaluated in this study. Wastewater effluents were obtained from a hospital within a six-month sampling period. After the samples were processed and analysed, *P. aeruginosa* was identified by polymerase chain reaction (PCR) by amplifying *OprI* and *OprL* genes. The Kirby–Bauer diffusion technique was employed to check the susceptibility profiles of *P. aeruginosa* which were further interpreted using CLSI guidelines. A total of 21 resistance genes were investigated among the isolates. The sum of 81 positive *P. aeruginosa* were isolated in this study. This study’s mean count of *Pseudomonas aeruginosa* ranged from 2.4 × 105 to 6.5 × 105 CFU/mL. A significant proportion of the isolates were susceptible to imipenem (93%), tobramycin (85%), norfloxacin (85%), aztreonam (70%), ciprofloxacin (51%), meropenem (47%), levofloxacin (43%), and gentamicin (40%). Meanwhile, a low susceptibility was recorded for amikacin and ceftazidime. The overall multiple antibiotics resistance index (MARI) ranged from 0.3 to 0.9, with 75% of the multidrug-resistant isolates. The assessment of β-lactam-resistant genes revealed *bla_OXA-1_* (3.7%) and *bla_SHV_* (2.4%). The frequency of carbapenem genes was 6.6% for *bla_IMP_*, 6.6% for *bla_KPC_*, 6.6% for *bla_oxa-48_*, 2.2% for *bla_NDM-1_*, 2.2% for *bla_GES_*, and 2.2% for *bla_VIM_*. Of the aminoglycoside genes screened, 8.6% harboured *strA*, 11.5% harboured *aadA*, and 1.5% harboured *aph(3)-Ia(aphA1).* Only one non-β-lactamase gene (*qnrA*) was detected, with a prevalence of 4.9%. The findings of this study revealed a high prevalence of multidrug-resistant *P. aeruginosa* and resistance determinants potentially posing environmental health risks.

## 1. Introduction

*Pseudomonas aeruginosa (P. aeruginosa*) is a common opportunistic pathogen with increased mortality and morbidity in immunocompromised individuals. It is commonly associated with nosocomial infection, high mortality and morbidity rates, and high treatment costs [1]. One of the primary contributors to mortality from Gram-negative septicaemia is *Pseudomonas aeruginosa* infection, which colonizes the lower respiratory and gastrointestinal tracts. Antibiotic-resistant pathogens of hospital origin pose an increased burden on the healthcare system and worldwide financial costs [2]. ESKAPE pathogens are the leading cause of hospital-acquired infections globally, with limited alternative treatment options due to their resistance mechanisms. The rate of collapse due to antibiotics administration, either in single or combination usage, increases the possibility of antibiotics with little or no effectiveness in the future [3,4].

Antibiotic-resistant *P. aeruginosa* is channelled into the natural environment through sewage disposals, fresh water, open ocean, and hospital effluents. Hospital effluents are one of the most common reservoirs of *P. aeruginosa,* characterized by inherent multidrug resistance with several strains resistant to various antibiotics [5]. Treating infectious diseases using antibiotics in hospitals promotes the spread of antibiotic-resistant bacteria in the environment due to antibiotic discharge and pharmaceutical wastes and chemicals [6]. Therefore, resistance is acquired through direct contact with sewage and effluents associated with resistant *P. aeruginosa* isolates [7]. Multidrug-resistant *P. aeruginosa* (MDRPA) discharge is, thus, increasingly perceived as a common driver in the global spread of antimicrobial resistance and calls for urgent public health action [8].

*P. aeruginosa* has been known to resist numerous antibiotics due to its outer membrane’s low permeability, multidrug efflux pumps, and biofilm formation [9]. The burden of resistant genes of distinct bacterial pathogens in hospital wastewater is a public health concern [6]. Mismanagement and abuse of antibiotics by humans stimulate the rapid escalation of genes associated with resistance in hospital wastewater, resulting in the rise of drug-resistant organisms in hospitals [10]. One of the mechanisms of *P. aeruginosa’s* resistance to antimicrobials is the production of β-lactamase enzymes like extended-spectrum β-lactamases (ESBLs) and AmpC and Metallo β-lactamases (MBLs) [11]. ESBLs hydrolyse penicillin, cephalosporins, and aztreonam. Meanwhile, AmpC β-lactamases hydrolyse cephalosporins and cephamycins and resist inhibition by clavulanate, sulbactam, and tazobactam. MBLs hydrolyse carbapenems and other β-lactams [12]. Carbapenems are effective against penicillin and cephalosporin-resistant Gram-negative bacteria [13]. The most important mechanism of carbapenem resistance in *P. aeruginosa* is the acquisition of genes encoding MBLs [14]. Class A ESBL enzymes such as *blaVEB, blaSHV*, *bla_CTX-M_, bla_PER_, bla_GES_*, and *bla_TEM_* are common among *P. aeruginosa* and *bla_OXA-1_*-type ESBL enzymes of class D [15]. The acquisition of new resistance genes by *P. aeruginosa* is intensified by its distribution into aquatic ecosystems [16]. Metagenomic investigation of untreated sewage used to characterize the bacterial resistome revealed significant changes in the diversity and abundance of antimicrobial resistance (AMR) genes [17,18].

There is a knowledge gap regarding the health risks associated with *P. aeruginosa* in hospital wastewater in South Africa. However, there is a need to evaluate the susceptibility patterns of hospital effluents before discharge to curb the increased health risk. As a result, this research aims to assess the prevalence, antibiogram profile, and burden of resistance determinants of *P. aeruginosa* isolated from hospital wastewater in South Africa.

## 2. Results

### 2.1. Colony Enumeration and Prevalence of P. aeruginosa

For the six-month sampling period (April-September), the average colony counts of *P. aeruginosa* ranged from 2.4 × 10^5^ to 6.5 × 10^5^ CFU/mL. Out of 144 presumptive isolates, 81 (56.25%) were confirmed by PCR assay as *P. aeruginosa.*

### 2.2. Antibiotic Susceptibility Patterns of Confirmed P. aeruginosa Isolates

**Figure 1:**
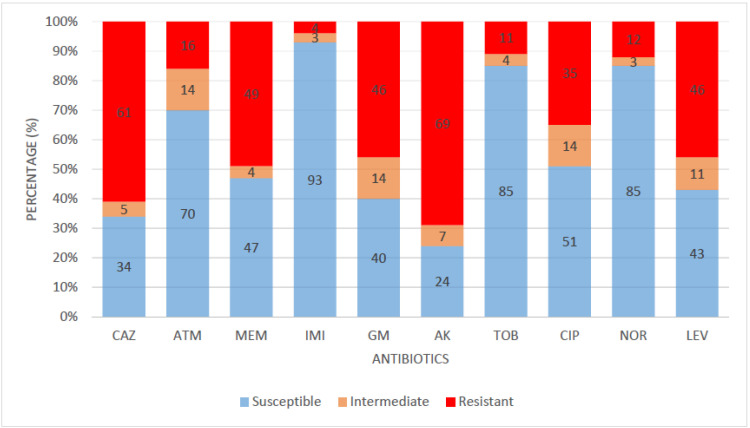
**Susceptibility pattern of *P. aeruginosa* isolates.** KEY: CAZ—ceftazidime, ATM—aztreonam, MEM—meropenem, IMI—imipenem, GM—gentamicin, AK—amikacin, TOB—tobramycin, CIP—ciprofloxacin, NOR—norfloxacin, LEV—levofloxacin.

All 81 confirmed *P. aeruginosa* isolates were evaluated for susceptibility to 10 panels of antibiotics in five antimicrobial classes. Imipenem had the highest sensitivity rate of 93%; a high percentage of the isolates were also susceptible to norfloxacin (85%), tobramycin (85%), aztreonam (70%), and ciprofloxacin (51%). Meanwhile, a lower sensitivity rate (below 50%) was observed against meropenem (47%), levofloxacin (43%), gentamicin (40%), ceftazidime (34%), and amikacin (24%). The susceptibility profile of the confirmed *P. aeruginosa* isolates is shown in Figure 1.

### 2.3. MDR and MARI Analysis

More than half—61 (75%)—of the phenotypically resistant *P. aeruginosa* isolates were resistant to three or more antibiotics classes, hence their classification as multidrug-resistant. The multiple antibiotics resistance index (MARI) of *P. aeruginosa* isolates ranged from 0.3 to 0.9. The MAR indices are shown in Table 1.

### 2.4. Distribution of Resistance Genes among P. aeruginosa Isolates

As shown in Table 2, among the 81 isolates resistant to β-lactams, 3.7% and 2.4% harboured *bla_oxa-1-like_* and *bla_SHV_* genes, respectively. The 69 aminoglycoside-resistant isolates tested had an overall frequency of *strA*, *aadA*, and *aph(3)-Ia(aphA1)^a^* in the following proportions: 8.6%, 11.5%, and 1.4%, respectively. Quinolone resistance gene *qnrA* was observed in 4.9% of the 81 quinolone-resistant isolates. Additionally, some of the isolates possessed some carbapenem genes as follows: *bla_IMP_* (6.6%), *bla_VIM_* (2.2%), *bla_KPC_* (6.6%), *bla_NDM-1_* (2.2%), *bla_OXA-48_* (6.6%), and *bla_GES_* (2.2%). None of the isolates harboured *bla_CTX-M-1_*, *bla_CTX-M-2_*, *bla_CTX-M-9_*, *bla_TEM_, bla_VEB_*, *bla_PER_*, *FOX*, *ACC*, *MOX*, *DHA*, *CIT*, *EBC*, *qnrB*, *qnrS,* or *aac(3)-IIa(aacC2)^a^* as investigated in this study.

## 3. Discussion

The widespread release of antibiotics and resistant bacteria into the environment through hospital effluents is an alarming public health concern. Hospital wastewater effluents serve as a primary source of antibiotics spread, hence the presence of antibiotics in various settings, such as domestic and hospital sewage [19]. These wastewater effluents are not effectively treated before they are released into nearby rivers. The importance of water bodies as a route for spreading bacteria has been acknowledged, and this spread is often associated with effluents from hospitals, animal farmland, and municipal sewage systems [20]. Contaminants found in hospital effluents usually resist water treatment, thereby being disseminated into nearby aquatic environments [21]. Antibiotics, major contaminants in wastewater effluents, are released into marine environments, disrupting ecosystems and posing a higher health risk when residents depend on rivers for irrigation, drinking, and recreational activities. Antibiotics resistance is a global menace to humans and animals [22]. Our investigation’s amplification of the *OprI* and *OprL* genes provided evidence of *P. aeruginosa.* The outer membrane lipoprotein *OprI* appears in two primary forms: a bound form, connected to a cell wall peptidoglycan, and a free form, lacking anchoring. The outer membrane protein *OprL* in *P. aeruginosa* is essential in the organism’s resistance to antibiotics and antiseptics [23]. The resistance stress of nosocomial pathogens such as *P. aeruginosa* isolated from untreated hospital wastewater was assessed in this study by analysing the antibiogram profile and resistance genes.

This study revealed the burden of *P. aeruginosa* in the study area with a prevalence of 56.25% (81/144). The average prevalence rate of *P. aeruginosa* recovered in this study could be due to the nutritional value of the hospital effluents and increased hospital-related infections. The prevalence of *P. aeruginosa* reported in this study is similar to that reported in another study where 72 *P. aeruginosa* isolates were obtained from hospital effluents [6]. Other studies have also reported reduced incidences of 26.04%, 20.83%, 14.58%, 18.75%, and 19.79% [7]. This study’s mean count of *Pseudomonas aeruginosa* ranged from 2.4 × 10^5^ to 6.5 × 10^5^ CFU/mL. During the sampling period, there was an observable progression in *Pseudomonas aeruginosa* count in May and July, while April, June, August, and September experienced a steady decline. Another report recorded a similar mean *Pseudomonas aeruginosa* population density ranging from 1.7 × 10^5^–6.1 × 10^5^ CFU/mL [7].

Most bacteria of clinical and environmental origin are mainly inhibited by antibiotics, whose consequence is severe therapy frustration. The confirmed isolates in our study revealed high sensitivity to the following classes of antibiotics: fluoroquinolones, carbapenems, aminoglycosides, and monobactams. The most effective drug in this study is imipenem (96%), a carbapenem with an occurrence rate of 96%. Carbapenem is a β-lactam antibiotic that has demonstrated efficacy for extended-spectrum β-lactamase-producing bacteria. Carbapenems are regarded as a last-resort antibiotic for treating illnesses brought on by multidrug-resistant Gram-negative bacteria. Carbapenems inhibit the peptidase domain of PBPs and also hinder peptide cross-linking. The capacity of carbapenems to bind to various PBPs is crucial to their efficiency. A similar high susceptibility of *Pseudomonas aeruginosa* against imipenem was reported in other studies [7,24]. Meanwhile, meropenem, also under the carbapenem class of antibiotics, showed a lower sensitivity rate of 47%. Regarding the fluoroquinolone class of antibiotics, 85%, 51%, and 43% of confirmed *Pseudomonas aeruginosa* isolates were susceptible to norfloxacin, ciprofloxacin, and levofloxacin. The fluoroquinolones inhibit type II DNA topoisomerases (gyrases) required for synthesizing bacterial mRNAs (transcription) and DNA replication. A study in South Africa also reported a similar sensitivity rate of 89% to norfloxacin [25]. The aminoglycosides, which include tobramycin, gentamicin, and amikacin, exhibited high sensitivity to the isolates. Aminoglycosides are known to reduce the growth of bacteria by blocking protein synthesis with high affinity to a ribosomal RNA active site in the 30S ribosome. A similar study reported a sensitivity rate of 93% against tobramycin [25]. Monobactams act mainly by inhibiting penicillin-binding protein 3 (PBP3), interfering with peptidoglycan synthesis and cell division. Aztreonam, a monobactam, was sensitive in this study in 70% of the *Pseudomonas aeruginosa* isolates. Ceftazidime and amikacin recorded a low sensitivity pattern among the isolates. Antibiotics resistance is a severe challenge that calls for urgent attention. The resistance of *P. aeruginosa* to ceftazidime is consistent with another survey [26]. Intriguingly, none of the isolates were resistant to all tested antibiotics.

MARI measures the health risks resulting from drug resistance spread in a given environment. The MARI range of 0.3–0.9 observed in this study is greater than the limit of >0.2, which shows that the isolates emanated from habitats harbouring numerous antibiotics. The value also indicates that chromosomes or plasmids enable antibiotics’ mechanisms. The MARI result in this study implies the environmental burden of antibiotic use and potential antibiotic pressure [7]. A similar MARI ranging from 0.33–0.89 was reported in a previous study [7]. Findings from *P. aeruginosa* isolated from abattoirs also had a MARI range of 0.4 to 0.8, similar to this study [9]. In this study, about 75% of the isolates were resistant to three or more classes of antibiotics, hence their classification as multidrug-resistant. Furthermore, among the MDR isolates, 14.8% were resistant to three antibiotics, 11.1% to four antibiotics, 14.8% to five antibiotics, 14.8% to six antibiotics, 12.3% to seven antibiotics, 4.9% to eight antibiotics, and 2.5% to nine antibiotics. Meanwhile, 25% of the *P. aeruginosa* isolates were resistant to less than three antibiotics.

*P. aeruginosa* acquires resistance traits by transferring genes between humans, animals, and the environment [27]. Production of *β*-lactamases is one of the resistance mechanisms of *β*-lactam antibiotics in *P. aeruginosa*, which inactivates the drugs, thereby mitigating the effect of antibiotics therapy [28]. Production of carbapenemase enables resistance to cephalosporins and monobactams. This study screened 21 resistance genes among *P. aeruginosa* isolates that showed varied resistance rates to the antibiotics tested. The aminoglycoside genes had the highest occurrence in this study, followed closely by the carbapenem genes. Among the isolates resistant to aminoglycosides, 8.6% harboured *strA,* 11.5% harboured *aadA*, and 1.5% harboured *aph(3)-Ia(aphA1)*. The carbapenem genes *bla_IMP_*, *bla_KPC_*, and *bla_oxa-48_* have an exact prevalence of 6.6%; likewise, *bla_NDM-1_*, *bla_GES_*, and *bla_VIM_* have a common prevalence rate of 2.2%. This study also detected two β-lactamase genes, with *bla_OXA-1_* being the most predominant, with a prevalence rate of 3.7%, alongside *bla_SHV_* (2.4%). *QnrA* is the only quinolone gene isolated in this study, with a rate of 4.9%. This study’s high abundance of antimicrobial resistance genes indicates an environmental burden, increasing possible health risks.

## 4. Materials and Methods

### 4.1. Research Area

**Figure 2:**
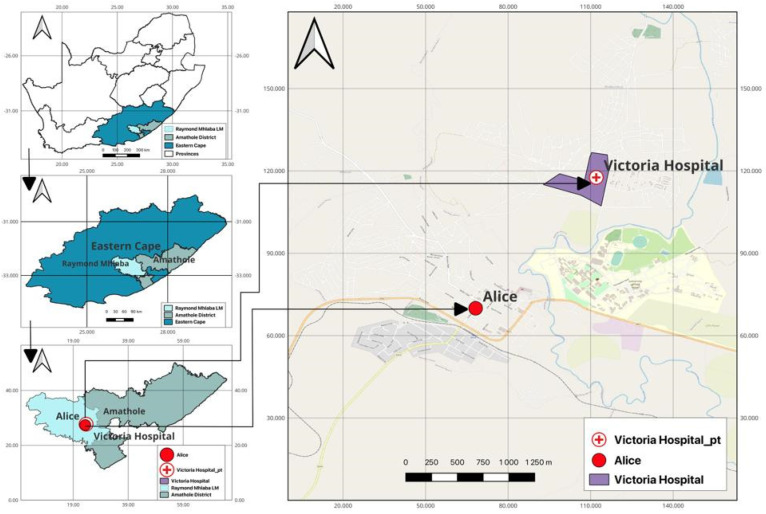
A map showing the study area.

The study was conducted between April 2021 and September 2021 at Victoria Hospital in Amathole District Municipality (ADM). The hospital is in Alice, a second small town in the Nkonkobe Municipality west of Amathole district (ADM), with an area coverage of 21,117 km^2^ and a population of approximately 880,790 people. Victoria Hospital is a provincial government-funded hospital in South Africa and the largest referral hospital for residents with numerous healthcare problems located in Raymond Mhlaba’s local municipality in ADM. ADM is situated in the heart of the Eastern Cape Province. It runs from the Fish River mouth on the Sunshine Coast. The Amathole Mountain Range borders ADM to the north. An estimated 72% of the municipality’s population lives in rural areas; 46% live in poverty, and 57.5% are out of a job [29]. The major economic sectors include community services, finance, manufacturing, trade, transport, and agriculture. The region is one of the indigents in South Africa and comprises mainly rural areas with substandard sanitary facilities. A map showing the sampling site and the neighbouring communities is shown in Figure 2.

### 4.2. Sample Collection

Wastewater effluent was aseptically obtained in triplicates from the sampling location in one-litre sterile glass bottles. An ice box was used to store the samples within 6 h of collection before being transported to the laboratory for analysis.

### 4.3. Isolation of Presumptive P. aeruginosa

A tenfold serial dilution of the wastewater samples was performed. A 100 mL aliquot was filtered through a membrane filter with a pore size of 0.45 μm (Sartorius, Goettingen, Germany) as described by the membrane filtration technique. The filtrate was aseptically placed on a plate with a cetrimide agar surface (Sigma-Aldrich, St. Louis, MO, USA) and incubated at 37 °C for 24 h. Each sample was analysed in triplicates. Distinct colonies with characteristic pyocyanin and pyoverdine pigments showing blue–green and yellow–green colours were considered presumptive for *P. aeruginosa*. The population densities of *P. aeruginosa* on cetrimide agar were counted as colony-forming per 100 mL (CFU/mL). The presumptive *P. aeruginosa* was purified on nutrient agar and stored for subsequent use.

### 4.4. DNA Extraction

The genomic DNA of presumptive isolates of *P. aeruginosa* was extracted by a boiling method, as previously described [30]. Two (2 mls) of the overnight cultures were placed into sterile Eppendorf tubes (Scientific Specialities, Inc., Lodi, CA, USA) and centrifuged (LASEC, Cape Town, South Africa) for 5 min at 13,500× *g* rpm. The supernatant was decanted, after which 500 µL of sterile distilled water was added and vortexed for 2 min using VELP, Scientifica. To lyse the cells, the suspension was heated at 100 °C for 10 min with MS2 Dri-Block DB.2A (Techne, London, UK) then placed on ice to cool. Subsequently, the suspension was centrifuged for 5 min at 13,500× *g* rpm, and the supernatant was carefully decanted into sterile Eppendorf tubes to be used as DNA templates. For further molecular analysis, the DNA templates were stored at −80 °C.

### 4.5. Confirmation of P. aeruginosa

Polymerase chain reaction (PCR) techniques were used to verify the presumptive *P. aeruginosa* isolates using species-specific primers. In a thermocycler (Bio-Rad, T100 thermal cycler Singapore, Hercules, CA, USA), a total reaction mixture of 25 μL was set up, consisting of 12.5 μL of master mix (Thermo Scientific, Waltham, MA, USA), 1 μL reach of already synthesized primer by Integrated DNA Technologies, USA, nuclease-free water (6.5 μL), and 4 μL of template DNA. Each amplicon (5 μL) was loaded in a 1.5% (*w*/*v*) horizontal agarose gel at 100 V for 45 min in 0.5× TBE buffer (pH-7.2). The gel was then stained with 4 μL ethidium bromide (0.5 μg/mL), and the size of each target gene was confirmed using a 100 bp DNA ladder (Fermentas, Vilnius, Lithuania). The PCR cycling condition was 94 °C (2 min), 94 °C (30 s), 62 °C (30 s), 72 °C (30 s), and 72 °C (10 min) × 35 cycles. A UV transilluminator (Alliance 4.7, UVITEC, Cambridge, UK) was used to examine the gel. The primer names and amplicon sizes are presented in Table 3.

### 4.6. Antibiotics Susceptibility Testing (AST)

According to Clinical and Laboratory Standards Institute (CLSI) guidelines (2011), AST was performed using the Kirby–Bauer disk diffusion method. The overnight bacterial broth (100–200 μL) in normal saline solution was adjusted to meet the 0.5 McFarland standard. Then, using a sterile glass spreader or sterile cotton swabs, 100 L was spread out on Muller–Hinton agar (Basingshike, Hampshire, England). Plates were impregnated with antimicrobial discs (Mast Diagnostics, Bootle, UK) using a disc-dispensing apparatus (Mast Diagnostics, UK). *P. aeruginosa* isolates were tested for susceptibility to a panel of ten antibiotics from five different classes. The antimicrobial discs used were imipenem (IMI, 10 μg), amikacin (AK, 30 μg), aztreonam (ATM, 30 μg), ceftazidime (CAZ, 30 μg), ciprofloxacin (CIP, 5 μg), tobramycin (TOB, 10 μg), gentamicin (GEN, 10 μg), meropenem (MEM, 10 μg), levofloxacin (LEV, 5 μg), and norfloxacin (NOR, 10 μg). The plates were then inverted for fifteen minutes of disc application and aerobic incubation at 37 °C for 24 h. Following that, the diameters of the zones of inhibition were measured and interpreted using the CLSI’s recommended criteria (CLSI (2018). *P. aeruginosa* ATCC 27853 was used for quality control. Multidrug resistance (MDR) was evaluated by opposition to antimicrobials in three or more classes [32].

### 4.7. Multiple Antibiotics Resistance Phenotype (MARP) and Multiple Antibiotics Resistance Index (MARI) Analysis

MARP patterns for multidrug-resistant *P. aeruginosa* isolates were evaluated by indicating the sequence of resistance to individual antibiotics. MARI was also assessed and interpreted for all positive *P. aeruginosa* isolates, as previously stated [33], MARI was calculated by dividing the number of antimicrobials to which the organisms were resistant (a) by the total number of antimicrobials (b) the organisms were exposed to. A MARI value greater than 0.2 indicates a possible environmental source of contamination due to high exposure to antibiotics, while a MARI value of ≤0.2 denotes minimal exposure to antibiotics usage.

### 4.8. Detection of Antimicrobial Resistance Genes

The resistant genes selected for screening were chosen due to their increased occurrence in resistant *P. aeruginosa* isolates. A total of 21 resistance genes responsible for extended-spectrum β-lactamases (*bla_CTX-M-1_*, *bla_CTX-M-2_*, *bla_CTX-M-9_*, *bla_OXA-1-like_*, *bla_TEM_*, *bla_SHV_*, *bla_VEB_*, *bla_PER_*), carbapenems (*bla_GES_*, *bla_OXA-48_ like*, *bla_NDM-1_*, *bla_IMP_*, *bla_VIM_*, *bla_KPC_*), quinolones (*qnrA*, *qnrB*, *qnrS*), and aminoglycosides (*aac(3)-IIa(aacC2)^a^*, *aadA*, *strA*, *aph(3)-Ia(aphA1)^a^)* were assessed in phenotypically resistant *P. aeruginosa*. Simplex, duplex, and multiplex PCR techniques were used to screen for these genes using specific primers and cycling conditions, as listed in Supplementary Materials Table S1. Agarose gel electrophoresis was used to examine the PCR products.

## 5. Conclusions

*P. aeruginosa* is a significant cause of hospital-acquired infection with severe disease risk. Hospital effluents are reservoirs of resistant *P. aeruginosa* isolates. There is a continuous and rapid spread of resistant *P. aeruginosa* in hospital effluents, causing unrest in aquatic ecosystems. Our research found that untreated hospital wastewater contained a substantial clinical pathogen (*P. aeruginosa*), which might potentially be released into the environment. Considering the high sensitivity of some of the isolates, most of the tested antibiotics can be administered for therapeutic purposes, excluding ceftazidime and amikacin. The capacity of *P. aeruginosa* to resist antimicrobial actions results in its spread and survival in the environment and, hence, its difficult elimination. Drug abuse and selective drug pressure are familiar drivers of antibiotics resistance. This research revealed antibiotic determinants’ burden in the study area. These resistance genes allow resistance to antimicrobials and result in unproductive treatment measures. This study is limited by the inability to sample more nosocomial pathogens and perform clonality analysis. Therefore, there is a need for proper surveillance and monitoring of hospital effluent discharge to circumvent the continuous dissemination and proliferation of resistant bacteria. Hospital wastewater should be adequately treated and be within the recommended safe standard before disposal.

## Figures and Tables

**Table 1:** MAR indices of resistant *P. aeruginosa* isolates.

No. of Antibiotics	MAR Phenotypes	No. of Phenotypes	MAR Index
3	AK-CAZ-ATM	1	0.3
	AK-CAZ-MEM	1	0.3
	AK-ATM-LEV	1	0.3
	AK-CAZ-LEV	1	0.3
	AK-CAZ-MEM	1	0.3
	AK-MEM-GM	1	0.3
	MEM-LEV-GM	1	0.3
	AK-CAZ-CIP	2	0.3
	CAZ-LEV-CIP	1	0.3
	AK-CAZ-GM	1	0.3
	MEM-GM-CIP	1	0.3
4	GM-AK-CAZ-ATM	1	0.4
	AK-CAZ-IMI-LEV	1	0.4
	AK-CAZ-LEV-CIP	1	0.4
	GM-AK-LEV-MEM	3	0.4
	GM-AK-MEM-ATM	2	0.4
	GM-AK-CAZ-MEM	1	0.4
5	GM-AK-LEV-CAZ-MEM	5	0.5
	GM-AK-LEV-MEM-CIP	1	0.5
	GM-AK-CAZ-MEM-CIP	2	0.5
	GM-AK-LEV-MEM-ATM	1	0.5
	GM-AK-LEV-CIP-ATM	1	0.5
	GM-LEV-CAZ-MEM-TOB	1	0.5
	GM-AK-LEV-MEM-NOR	1	0.5
6	CIP-IMI-LEV-CAZ-MEM-TOB	1	0.6
	CIP-LEV-CAZ-MEM-GM-AK	3	0.6
	CIP-LEV-CAZ-GM-AK-NOR	1	0.6
	CIP-LEV-CAZ-AK-NOR-ATM	1	0.6
	CIP-LEV-CAZ-GM-AK-ATM	2	0.6
	CIP-LEV-CAZ-GM-AK-IMI	2	0.6
	CAZ-MEM-TOB-GM-AK-ATM	1	0.6
	CIP-LEV-MEM-GM-AK-ATM	1	0.6
7	GM-CIP-AK-CAZ-MEM-ATM-TOB	1	0.7
	GM-CIP-AK-CAZ-MEM-ATM-LEV	1	0.7
	GM-CIP-AK-CAZ-MEM-LEV-NOR	2	0.7
	GM-CIP-AK-CAZ-ATM-LEV-NOR	1	0.7
	GM-CIP-AK-CAZ-TOB-LEV-NOR	1	0.7
	GM-AK-CAZ-MEM-ATM-TOB-LEV	1	0.7
	GM-CIP-AK-CAZ-MEM-ATM-TOB	1	0.7
	CIP-AK-CAZ-MEM-ATM-LEV-NOR	1	0.7
	GM-CIP-AK-CAZ-MEM-ATM-LEV	1	0.7
8	GM-CIP-AK-LEV-CAZ-MEM-ATM-TOB	2	0.8
	GM-CIP-AK-LEV-CAZ-MEM-ATM-NOR	1	0.8
	GM-CP-AK-LEV-CAZ-ATM-TOB-NOR	1	0.8
9	GM-CIP-AK-IMI-LEV-NOR-CAZ-MEM-TOB	1	0.9
	GM-CIP-AK-LEV-NOR-CAZ-MEM-TOB-ATM	1	0.9

**KEY:** CAZ—ceftazidime, ATM—aztreonam, MEM—meropenem, IMI—imipenem, GM—gentamicin, AK—amikacin, TOB—tobramycin, CIP—ciprofloxacin, NOR—norfloxacin, LEV—levofloxacin.

**Table 2:** Distribution of resistance genes among *P. aeruginosa* isolates.

Antibiotics Resistance Genes.	Total Positive (%)
**β** **-Lactams (*n* = 81 isolates screened)**	
*bla_SHV_*	2 (2.4)
*bla_OXA-1 LIKE_*	3 (3.7)
**Carbapenem (*n* = 45 tested)**	
*bla_IMP_*	3 (6.6)
*bla_VIM_*	1 (2.2)
*bla_KPC_*	3 (6.6)
*bla_NDM-1_*	1 (2.2)
*bla_OXA-48_*	3 (6.6)
*bla_GES_*	1 (2.2)
**Aminoglycoside (*n* = 69 tested)**	
*strA*	6 (8.6)
*aadA*	8 (11.5)
*aph(3)-Ia(aphA1)^a^*	1 (1.4)
**Quinolones (*n* = 81 tested)**	
*qnrA*	4 (4.9)

**Table 3:** Primer sequences for the molecular identification of *P. aeruginosa*.

Target Strain	Target Gene	Primer Sequence (5^1^–3^1^)	Product Size (bp)	Reference
*Pseudomonas* genus	*OprI*	F:ATGAACAACGTTCTGAAATTCTCTGCT R:CTTGCGGCTGGCTTTTTCCAG	249	[31]
*Pseudomonas aerugionsa*	*OprL*	F:ATGGAAATGCTGAAATTCGGC R: CTTCTTCAGCTCGACGCGACG	504	[31]

## Data Availability

Further information will be provided on request.

## Supplementary Materials

The following supporting information can be downloaded at: https://www.mdpi.com/article/10.3390/antibiotics12101517/s1, Table S1: Primer sequences of targeted resistance genes with their respective amplicon sizes and PCR cycling conditions.
