# Supplementary material for: Antibiogram Profile and Detection of Resistance Genes in Pseudomonas aeruginosa Recovered from Hospital Wastewater Effluent
Source: Antibiotics (Basel). 2023 Oct 6;12(10):1517. doi: 10.3390/antibiotics12101517 (PMC10604762; doi:10.3390/antibiotics12101517)
Supplement: Supplementary file 1 [file antibiotics-12-01517-s001.zip › antibiotics-2573331-supplementary.pdf]

antibiotics

MDPI

Supplementary Materials

Table S1. Primer sequences of targeted resistance genes with their respective amplicon sizes and PCR cycling conditions.

Gene Classification	Target Gene	Primer Sequence (5^{1}-3^{1})	PCR Cycling Condition	Product Size (bp)	References
β-lactam<br>*bla*$_{\text{CTX-M 1}}$	MultiCTXMGp1_for<br>MultiCTXMGp1-2_rev	TTAGGAARTGTGCCGCTGYA[b]<br>CGATATCGTTGGTGGTRCCAT[b]	94 °C (10 min), 94 °C (40 s), 60 °C (40 s), 72 °C (1 min), 72 °C (7 min) × 30 cycles	688	[32]
bla$_{\text{CTX-M-2}}$	MultiCTXMGp2_for<br>MultiCTXMGp1-2_rev	CGTTAACGGCACGATGAC<br>CGATATCGTTGGTGGTRCCAT[b]	''94 °C (10 min), 94 °C (40 s), 60 °C (40 s), 72 °C (1 min), 72 °C (7 min) × 30 cycles"	404	[32]
bla$_{\text{CTX-M-9}}$	MultiCTXMGp9_for<br>MultiCTXMGp9_rev	TCAAGCCTGCCGATCTGGT<br>TGATTCTCGCCGCTGAAG	''94 °C (10 min), 94 °C (40 s), 60 °C (40 s), 72 °C (1 min), 72 °C (7 min) × 30 cycles"	561	[32]
bla$_{\text{OXA-1-like}}$	MultiTSO-O_for<br>MultiTSO-O_rev	GGCACCAGAT-TCAACTTTCAAG<br>GACCCCAAGTTTCCTG-TAAGTG	''94 °C (10 min), 94 °C (40 s), 60 °C (40 s), 72 °C (1 min), 72 °C (7 min) × 30 cycles"	564	[32]
bla$_{\text{SHV}}$	MultiTSO-S_for<br>MultiTSO-S_rev		''94 °C (10 min), 94 °C (40 s), 60 °C (40 s), 72 °C (1 min), 72 °C (7 min) × 30 cycles"	713	[32]
		AGCCGCTTGAG-CAAATTAAAC ATCCCG-CAGATAAATCACCAC	''94 °C (10 min), 94 °C (40 s), 60 °C (40 s), 72 °C (1 min), 72 °C (7 min) × 30 cycles"		
bla$_{\text{TEM}}$	MultiTSO-T_for<br>MultiTSO-T_rev			800	[32]
		CATTTCCGTGTCGCCCTTATTC<br>CGTTCATCCATAGTT-GCCTGAC	''94 °C (10 min), 94 °C (40 s), 60 °C (40 s), 72 °C (1 min), 72 °C (7 min) x 30 cycles"		
bla$_{\text{VEB}}$	MultiVEB_for<br>MultiVEB_rev		''94 °C (10 min), 94 °C (40 s), 60 °C (40 s), 72 °C (1	648	[32]

		CATTTCCCGATGCAAAGCGT<br>CGAAGTTTCTTTGGACTCTG	min), 72 °C (7 min) × 30 cycles”		
bla$_{PER}$	MultiPER_for<br>MultiPER_rev		’’94 °C (10 min), 94 °C (40 s), 57 °C (40 s), 72 °C (1 min), 72 °C (7 min) × 30 cycles”	520	[32]
		GCTCCGATAATGAAAGCGT<br>TTCGGCTTGACTCGGCTGA		399	[32]
bla$_{GES}$	MultiGES_for<br>MultiGES_rev				
		AGTCGGCTAGACCGGAAAG<br>TTTGTCCGTGCTCAGGAT			
		GATGGTGTTTGGTCGCATA<br>CGAATGCGCAGCACCAG			
Carbapenem<br>*bla*$_{VIM}$			’’94 °C (10 min), 94 °C (40 s), 55 °C (40 s), 72 °C (1 min), 72 °C (7 min) × 30 cycles”	390	[32]
	MultiVIM_for[c]<br>MultiVIM_rev[c]	TTGACACTCCATTTACDG[b]<br>GATYGAGAATTAAGCCA-CYCT[b]			
bla$_{IMP}$			’’94 °C (10 min), 94 °C (40 s), 55 °C (40 sec), 72 °C (1 min), 72 °C (7 min) x 30 cycles”	139	[32]
	MultiIMP_for<br>MultiIMP_rev	TTGGTGGCATCGATTATCGG<br>GAGC ACTT CTTT TGTG ATGG C			
bla$_{OXA-48-like}$	bla$_{OXA-48-like-F}$<br>bla$_{OXA-48-like-R}$		’’94 °C (10 min), 94 °C (40 s), 57 °C (40 s), 72 °C (1 min), 72 °C (7 min) × 30 cycles”	281	[32]
bla$_{NDM-1}$	*bla*$_{NDM-1}$-F<br>*bla*$_{NDM-1}$-R	AAAACGGCAAGAAAAA-GCAG<br>AAAACGGCAAGAAAAA-GCAG	’’94 °C (10 min), 94 °C (40 s), 57°C (40 s), 72 °C (1 min), 72 °C (7 min) × 30 cycles”	231	[32]
bla$_{KPC}$	*bla*$_{KPC}$-F<br>*bla*$_{KPC}$-R	AAAACGGCAAGAAAAA-GCAG<br>AAAACGGCAAGAAAAA-GCAG	’’94 °C (10 min), 94 °C (40 s), 55 °C (40 s), 72 °C (1 min), 72 °C (7 min) × 30 cycles”	301	[32]

**Quinolones**	*qnrA*	ATTTCTCACGCCAGGATTTG GATCGGCAAAGGTTAGGTCA	''94 °C (10 min), 94 °C (45 s), 53 °C (45 s), 72 °C (1 min), 72 °C (7 min) × 32 cycles"	448	[33]
	qnrB	GATCGTGAAAGCCAGAAAGG ACGATGCCTGGTAGTTGTCC	''94 °C (10 min), 94 °C (45 s), 53 °C (45 s), 72 °C (1 min), 72 °C (7 min) × 32 cycles"	469	[33]
	qnrS	ACGACATTCGTCAACTGCAA TAAATTGGCACCCTGTAGGC	''94 °C (10 min), 94 °C (45 s), 53 °C (45 s), 72 °C (1 min), 72 °C (7 min) × 32 cycles"	417	[33]
**Aminoglyco-sides**	*aac(3)-IIa(aacC2)*[a]	F:CGGAAGGCAATAACGGAG R:TCGAACAGGTAGCACTGAG	''94 °C (5 min), 94 °C (30 s), 50 °C (30 s), 72 °C (1.30 min), 72 °C (5 min) × 30 cycles"	428	[34]
	aadA	F:GTGGATGGCGGCCTGAA-GCC R:AATGCCCAGTCGGCAGCG	''94 °C (4 min), 94 °C (45 s), 50 °C (45 s), 72 °C (45 s), 72 °C (7 min) × 30 cycles"	525	
	strA	F:CTTGGTGATAACGGCAATTC R:CCAATCGCAGATAGAAGGC	''94 °C (4 min), 94 °C (45 s), 50 °C (45 s), 72 °C (45 s), 72 °C (7 min) × 30 cycles"	348	[35]
	aph(3)-Ia(aphA1)[a]	F:ATGGGCTCGCGATAATGTC R:CTCACCGAGGCAGTTCCAT	''94 °C (5 min), 94 °C (30 s), 50 °C (30 s), 72 °C (1.30 min), 72 °C (5 min) × 30 cycles"	110	[34]
